# Comment on Sarathkumara et al.: Exposure to Hantavirus is a Risk Factor Associated with Kidney Diseases in Sri Lanka: A Cross Sectional Study

**DOI:** 10.3390/v11121147

**Published:** 2019-12-11

**Authors:** W. Katherine Yih, Martin Kulldorff, Jessica H. Leibler, David J. Friedman, Daniel R. Brooks

**Affiliations:** 1 Department of Population Medicine, Harvard Medical School and Harvard Pilgrim Health Care Institute, Boston, MA 02215, USA; 2 Division of Pharmacoepidemiology and Pharmacoeconomics, Department of Medicine, Harvard Medical School and Brigham and Women’s Hospital, Boston, MA 02115, USA; martin_kulldorff@hms.harvard.edu; 3 Department of Environmental Health, Boston University School of Public Health, Boston, MA 02118, USA; jleibler@bu.edu; 4 Division of Nephrology, Beth Israel Deaconess Medical Center, Harvard Medical School, Boston, MA 02215, USA; dfriedma@bidmc.harvard.edu; 5 Department of Epidemiology, Boston University School of Public Health, Boston, MA 02118, USA; danbrook@bu.edu

In a recent paper, Sarathkumara et al. [1] concluded that hantavirus infection might be an important risk factor for development of chronic kidney disease of unknown etiology (CKDu) in Sri Lanka. We do not contest this possibility. However, it may be that these findings are confounded by age, sex, occupation, or other co-variates, any of which could plausibly be related to both hantavirus exposure and CKDu. We would urge the authors to use their rich and valuable dataset to conduct a multivariable logistic regression analysis with CKDu as the dependent variable and *all appropriate co-variates included in the model*, as in a recent analysis of CKDu and leptospirosis [2]. Whether or not the results are null, such an analysis would constitute a significant contribution to the field, as the etiology of CKDu has not yet been elucidated despite serious, concerted research efforts.
